# Male or Female? It Depends on the Dose

**DOI:** 10.1371/journal.pbio.0040211

**Published:** 2006-05-23

**Authors:** Liza Gross

In most cases, a fertilized egg with two X chromosomes will become a girl and an XY egg will become a boy. Molecular analysis of the genomes of sex-reversed individuals identified the sex-determining region Y gene *(Sry)*, the primary sex-determining gene in mammals. The presence of an *Sry* gene normally drives male development. But when downstream elements of the sex-determining pathway are disrupted, chromosomal sex and sexual phenotype can be discordant. This phenomenon revealed a fatal flaw in a now-defunct Olympics rule requiring female athletes to submit to “gender verification” procedures that labeled XY females “imposters.”

Every embryonic gonad—at this point, neither ovary nor testis—harbors cells with the potential to choose either fate (male or female). Reflecting this noncommittal approach, both XX and XY gonads initially display similar expression patterns for the sex-determining genes *Sox9*, *Fgf9*, and *Wnt4*. If *Sry* does not intervene during a critical window in development, the cells will default to the ovarian pathway. Once *Sry* expression begins, other genes start to choose sides: *Sox9* and *Fgf9* become active only in male gonads, while *Wnt4* becomes active only in female gonads. Loss of *Fgf9* in XY mice leads to sex reversal; in XX mice, loss of *Wnt4* leads to partial testis development. Only when the protein products of *Sry*, *Sox9*, and *Fgf9* are all expressed together do male-specific Sertoli cells develop—and prod the developing gonad toward full male differentiation.

Clearly, *Fgf9* and *Wnt4* must somehow influence sex determination—but how? In a new study, Yuna Kim, Blanche Capel, and colleagues explored how *Sry*, *Sox9*, *Fgf9*, and *Wnt4* contribute to the initial events of sex determination in mice. By manipulating the activity of these signals, they show that *Wnt4* and *Fgf9* play a sexual tug of war. If *Wnt4* wins, the gonad differentiates into an ovary. If *Fgf9* wins, it turns into a testis. Living up to its name, *Sry* tips the balance in favor of *Fgf9*, by triggering expression of *Sox9*, and a male fate.

The researchers started by examining *Fgf9* expression during normal gonad development in the mouse. 11.5 days after conception, *Fgf9* was evenly distributed in the gonads of both sexes. By day 12, it was found only in XY gonads, and only in Sertoli cells within testis cords and cells near the surface of the gonad.

Seeing *Fgf9* expressed so early in testis differentiation suggested it might trigger *Sry* activity or expression, but the researchers found that this wasn't the case. If *Fgf9* doesn't regulate *Sry*, they reasoned, maybe it affects the male-specific up-regulation of *Sox9*, which is absent in XY mice lacking *Fgf9*. Adding *Fgf9* to XX cells isolated from 11.5-day-old gonads triggered increased *Sox9* expression. These findings showed that artificial expression of *Fgf9* can induce *Sox9* expression in single XX cells, but what about in the intact gonad?

In normal and mutant *Fgf9* XY mice, *Sox9* expression followed similar patterns for the first 24 hours after *Sry* was expressed. By 12.5 days, SOX9 proteins were no longer expressed in the mutant mice, and the cells that would normally have developed into Sertoli cells failed to organize into normal testis cords. These results, the researchers conclude, indicate that *Fgf9* isn't necessary to trigger *Sox9* expression, but instead is required to maintain *Sox9* expression in Sertoli precursor cells.

*Fgf9* expression depends on *Sox9*, and *Fgf9* returns the favor by maintaining *Sox9* expression in a positive feed-forward loop. The feed-forward loop between these genes allows the proliferation and expansion of Sertoli and other somatic cells in XY gonads. Without *Fgf9*, XY gonads showed a rapid drop in SOX9 protein levels and expressed few or no Sertoli cell markers. *Wnt4*, on the other hand, increased, suggesting it was repressed by *Fgf9*. Without the antagonistic effects of *Fgf9*, *Wnt4* predominates, disrupting the *Fgf9–Sox9* pathway and establishing the infrastructure necessary to become female. And the reverse holds true: reducing *Wnt4* levels in XX gonads allows FGF9 and SOX9 levels to increase—even in the absence of *Sry*—though other male factors appear to be required to establish testis development.

Altogether, these results suggest that sex is not determined by the flip of a genetic switch but by a dose-dependent interplay between opposing signals. The researchers propose that *Sry* initiates the male fate by triggering *Sox9* expression, which activates *Fgf9*, and sets off a feed-forward loop that produces a male, barring the interference of antagonistic signals. Whether these signals work within or between cells is not clear. It may be, for example, that *Fgf9* recruits other cells to the Sertoli pathway by triggering increased *Fgf9–Sox9* expression, or that *Fgf9* causes Sertoli cells to proliferate until they reach the critical mass needed to fend off *Wnt4* and secure the male pathway. Future studies can investigate the molecular mechanisms of *Fgf9* and *Wnt4*—and whether these competing pathways regulate the battle of the sexes in a wide range of vertebrates.

## 

**Figure:**
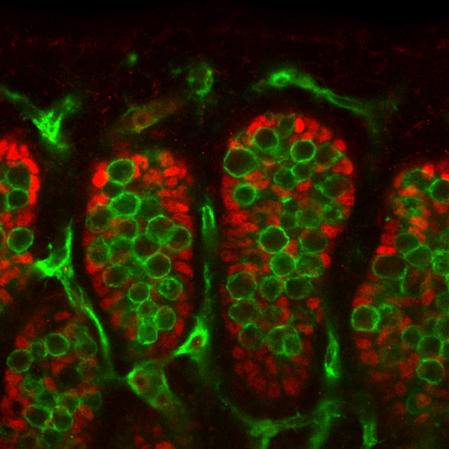
Fgf9 is critical to repress Wnt4 and establish the testis pathway during mamma lian sex determination. Sertoli cells (red), germ cells, and endothelial cells (green).

